# Key constituents for integration of care for children assisted with long-term home ventilation: a European study

**DOI:** 10.1186/s12887-020-1979-4

**Published:** 2020-02-15

**Authors:** Maria Brenner, Miriam P. O’Shea, Philip Larkin, Jay Berry

**Affiliations:** 1 School of Nursing & Midwifery, Trinity College Dublin University of Dublin, 24 D’Olier Street, Dublin 2, Ireland; 2 Institut Universitaire de Formation et de Recherche en Soins, Bureau 01/157, SV-A Secteur Vennes, Rte de la Corniche 10, CH-1010 Lausanne, Switzerland; 3 Department of Medicine and Division of General Pediatrics, Boston Children’s Hospital and Harvard Medical School, Boston, USA

**Keywords:** Access to care, Care coordination, Complex care needs, Family partnership, Governance, Long-term home ventilation, Palliative care, Quality of care, Respite care

## Abstract

**Background:**

The number of children requiring long-term home ventilation has consistently increased over the last 25 years. Given the growing population of children with complex care needs (CCNs), this was an important area of focus within the Models of Child Health Appraised (MOCHA) project, funded by the European Union (EU) under the Horizon 2020 programme. We examined the structures and processes of care in place for children with CCNs and identified key constituents for effective integration of care for these children at the community and acute care interface across 30 EU/ European Economic Area (EEA) countries.

**Methods:**

This was a non-experimental descriptive study with an embedded qualitative element. Data were collected by a Country Agent in each of the 30 countries, a local expert in child health services. Data were analysed using descriptive statistics and a thematic analysis was undertaken of the free text data provided.

**Results:**

A total of 27 surveys were returned from a possible 30 countries (90.0%) countries. One respondent indicated that their country does not have children on long-term ventilation (LTV) in the home, therefore, responses of 26 countries (86.7%) were analysed. None of the responding countries reported that they had all of the core components in place in their country. Three themes emerged from the free text provided: ‘family preparedness for transitioning to home’, ‘coordinated pathway to specialist care’ and ‘legal and governance structures’.

**Conclusions:**

While the clinical care of children on LTV in the acute sector has received considerable attention, the results identify the need for an enhanced focus on the care required following discharge to the community setting. The results highlight the need for a commitment to supporting care delivery that acknowledges the complexity of contemporary child health issues and the context of the families that become their primary care givers.

## Background

There is an increasing focus on the management of care of children with complex care needs (CCNs), defined as multidimensional health and social care needs, in the presence of a recognised medical condition or where there is no unifying diagnosis [1]. The provision of care closer to home has been identified internationally as an important objective for this population [2, 3]. This supports a child-centric approach to care within a health service that is increasingly focused on enhancing integrated care delivery. Given the growing population of children with CCNs, little is known about the structures and processes currently in place across the EU to support the care requirements of these children and their families. This was therefore an important area of focus within the Models of Child Health Appraised (MOCHA) project, funded by the EU under the Horizon 2020 programme. We examined the structures and processes of care in place for children with CCNs and identified key characteristics for effective integration of care for these children at the community and acute care interface across 30 European Union (EU)/ European Economic Area (EEA) countries. Three specific exemplars were chosen for examination in each of the following areas: traumatic brain injury, long-term ventilation (LTV), and intractable epilepsy. The exemplars were developed by a team of clinical and academic experts and ratified by the external advisory board to the project. The advisory board comprised of European medical, paediatric, and policy bodies, and civil society groups, including the youth subgroup of the European Patients’ Forum [4]. This paper specifically reports on the findings on the constituents for the effective care for children assisted with LTV in the home.

It is reported that the number of children requiring LTV has consistently increased over the last 25 years. This is supported by a study in the United Kingdom, from 1994 to 2010 which showed a 30-fold increase in prevalence between 1994 and 2010, from 0.2–6.7 per 100,000 [5], and more recent data available across the EU. This includes data from Italy showing a prevalence of 4.2 per 100,000 [6], and a report from Austria showing a prevalence of 7.4 per 100,000 [7]. This is supported by trends reported internationally. For example, the number of children receiving LTV at home in Canada increased from 2 in 1991 to 156 in December 2011. This study found a twofold increase in the number of invasive ventilation initiations in the second 10 years, *n* = 45 (2001–2011) compared to the first 10 years, *n* = 21 (1991–2000) [8]. Children are reliant on LTV for a variety of reasons which may include chronic lung disease due to prematurity, congenital airway malformations, hypoventilation syndrome, neuromuscular diseases, and spinal cord injuries [9]. Therefore, these children often have frequent readmissions to hospital, are cared for by numerous health and social care providers, and are resource intensive in terms of care delivery at home [10–13]. Data from studies that have investigated long-term outcomes for these children are scarce; those that exists indicate reduced long-term health related quality of life [14]. An examination of the structures and processes, and an exploration of the key constituents required for enhanced care, was therefore required to provide key foundational knowledge for the development of care services for these children and their families.

## Methods

### This was a non-experimental descriptive study with an embedded qualitative element

#### Survey

This is the first time that the care of children with CCNs was examined across the EU/EEA. The Standards for Systems of Care for Children and Youth with Special Health Care Needs [15] were adapted with the permission of the Lucile Packard Foundation. Detail of the adaptation of the standards for MOCHA can be found at www.childhealthservicemodels.eu The areas of care explored in this survey were: screening, assessment and referral; access to care; care coordination; community-based services; family-professional partnerships; and quality assurance. An exemplar on LTV (Table 1) and related questions were provided to each participant. Participants were offered an opportunity to include additional comments on key characteristics for effective integration of care for these children and their families.

**Table 1:** Exemplar for child assisted by long-term ventilation

Max is an 18 month old boy with a diagnosis of chronic lung disease due to bronchopulmonary dysplasia. Max was born at 26 weeks gestation weighing less than 1 kg. He had a diaphragmatic hernia, a gastrostomy tube placement at 3 months of age, and a Grade IV intraventricular haemorrhage requiring a cerebrospinal fluid ventricular shunt. Max has been ventilator dependent since he was born and is considered to have a life-threatening condition. A tracheostomy tube was placed at 6 weeks of age due to the need for ongoing ventilation. Max spent the first 3 months of his life in intensive care, followed by 4 months in a step-down/transitional care unit. At present Max has the following: impaired pulmonary function, developmental delay in fine and gross motor skills, and speech and language difficulties. His prognosis for weaning off the ventilator does not seem favourable at the moment and ideally he requires the healthcare input of the following healthcare professionals: community nurses, specialist consultants (respiratory, paediatrician, neurology), community general practitioner, pharmacist, speech and language therapist, physiotherapist, occupational therapist, social worker, dentist, home care nursing team and respite care services. He lives with his two sisters, aged 5 and 7 years, and his mother and father. He lives 120kms from the main children’s hospital and 40kms from his nearest regional hospital which has a small paediatric unit.	

### Data collection

Data were collected by a Country Agent (CA) in each of the 30 countries between July and December 2016. This was a key methodological feature of the MOCHA project, the remunerated retention in each country of a part-time CA – a local expert in child health services – who acted as the informant for obtaining data requested by the principal scientists in the project. The data was collected by the CA from local indigenous sources including health and social care experts, policy makers, health care governing bodies, and expert stakeholders including parents/guardians and advocacy groups. For more information about the country agents, please see the MOCHA website http://www.childhealthservicemodels.eu/partnerlisting/country-agents.

### Analysis

Data were analyzed using descriptive statistics and a thematic analysis was undertaken of the free text data provided. Attride-Stirling’s thematic analysis network [16] was applied to the textual data.

## Results

A total of 27 surveys were returned from a possible 30 countries (90.0%) countries. One of the 27 respondents indicated that their country did not have home LTV, therefore, responses of 26 countries (86.7%) were analysed.

### Structures and processes to support children assisted with LTV

The key findings from all domains surveyed are presented in Table 2. No country had all of the core components in place for all of the domains examined and there was therefore no discernible pattern overall in the results. It was reported that Italy had all of the structures and processes examined in place for four of the six domains: screening, assessment and referral; access to care; community-based services; and family-professional partnerships. Norway reported that it had all of the structures and processes in place for three domains: screening, assessment and referral; access to care; and care coordination. Three countries reported that they had the structures and processes in place for two of the six domains: Estonia - screening, assessment and referral and access to care; The Netherlands - care coordination and community-based care; and Croatia - community-based care and family-professional partnerships. A further seven countries reported that they had all the structures and processes in place for one of the domains, all of which were varied. The only similarity in this finding was that Denmark and Portugal had all core components in place for screening, assessment and referral, while the Czech Republic and Lithuania had all core components in place for access to care.

**Table 2:** Structures and processes to support children assisted with LTV

*Domain and Item*	*Number of countries %(n)*
**Screening and Assessment**	
Policies and procedures to support preventative screening, assessment and referral for routine developmental checks	46.2(12)
Mechanisms in place to document and communicate the results of assessments and screening to all the services who provide care to the child	46.2(12)
Mechanisms in place to support the communication of screening and assessment to parents/guardians	38.5(10)
**Access to Care**	
Mechanisms in place to identify all healthcare providers caring for the child	53.8(14)
Assistance with transport of the child to hospital appointments	30.8(8)
Policies or procedures in place to support the provision of linguistically appropriate information to the family	41.7(12)
Policies or procedures in place to support the provision of culturally appropriate information to the family	42.3(11)
**Care Coordination**	
Policies and procedures in place to support care coordination	53.8(14)
Discharge Planning Coordinator in place in paediatric departments/hospitals	42.(11)
Consultation with parents/ guardians in the development of personalised care plans	84.6(22)
Consultation with all healthcare professionals in the development of personalised care plans	73.1(19)
**Community Based Services and Supports**	
Family advocacy groups involved in making recommendations to home and community-based services	38.5(10)
Policies in place to support paediatric palliative care and end-of-life care	65.4(11)
Access to psychological support for parents/guardians and siblings	84.6(22)
**Family-Professional Partnerships**	
Family advocacy groups involved in the development of policies and procedures affecting the care of the child	46.2(12)
Parents/guardians included in national quality improvements	30.8(8)
Parents/guardians included in the review of patient / family information	38.5(10)
**Quality Assurance**	
Policies or procedures in place to support quality assurance for service providers	57.7 (15)
Data collected on the experience of care from the perspective of the parents/guardians	30.7 (8)
Data collected on the experience of care from the perspective of the siblings	11.5 (3)

### Facilitators of effective integration of care at the community and secondary care interface for a child assisted with LTV

Three global themes (GTs) emerged from the text provided by the CAs: ‘family preparedness for transitioning to home’, ‘coordinated pathway to specialist care’ and ‘legal and governance structures’.

### Family preparedness for transitioning to home

**Fig. 1:**
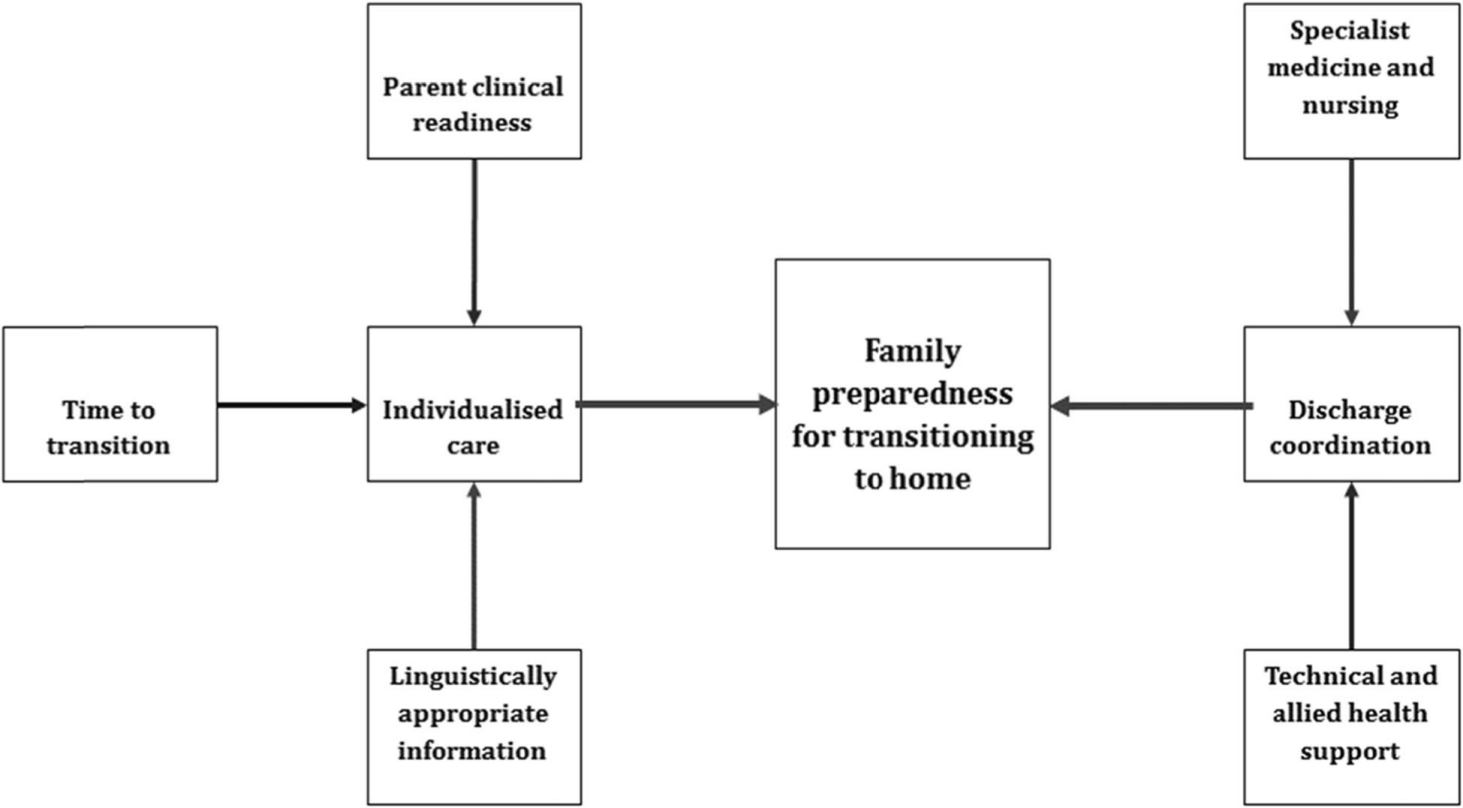
Emergence of global theme ‘family preparedness for transitioning to home’

The theme ‘family preparedness for transitioning to home’ describes the characteristics of effective integration of care at the community and acute care interface for children assisted with LTV as parents transition to becoming the child’s primary care giver in the home. This theme emerged from accounts from CAs on facilitators of a successful transition to home currently in place in their country and arose from two organising themes (OTs) ‘individualised care’ and ‘discharge coordination’ (Fig. 1). The OT ‘individualised care’ refers to the clinical readiness of parents to take care of their child on LTV following their discharge from hospital. Linguistically appropriate information was identified as a key constituent to effective individualised care, to support equity of care and optimum readiness for caring for the child at home. It was also suggested that parents are best supported in their transition to home when they have the opportunity to gradually increase their care input, according to their own perceived level of readiness to become the child’s primary caregiver. A phased step-down plan was identified as an appropriate way to facilitate this, whereby parents increase their input in to the clinical care of their child in an incremental manner.

*If parents feel more secure, the child comes to a step down unit, where the parents share a greater part of care themselves, but know they can always call someone for support. The last step in inpatient care before discharging, is the regular ward, where the parents look after the child more-or-less themselves. There are no time limits for the duration of the stay… Only when the parents feel safe and do well, and agree, the child will be discharged at home.* (CA Austria)

The second OT ‘discharge coordination’ emerged from data from a number of countries who provided examples of support offered to enhance family preparedness for transitioning to home. Central to this was the role of a Discharge Coordinator, who was identified as being the central point of communication and planning at the acute community interface, for specialist medical and nursing input, and for technical support and allied health input prior to discharge. In terms of specialist medical and nursing input, this refers to linking the acute care team with the community care team, to ensure the community care team would have a full picture of the clinical care needs of the child transitioning to home. In addition the discharge coordinator would ideally be responsible for ensuring all the technical support is appropriately planned and in place prior to the child’s discharge from hospital.

### Coordinated pathway to specialist services

**Fig. 2:**
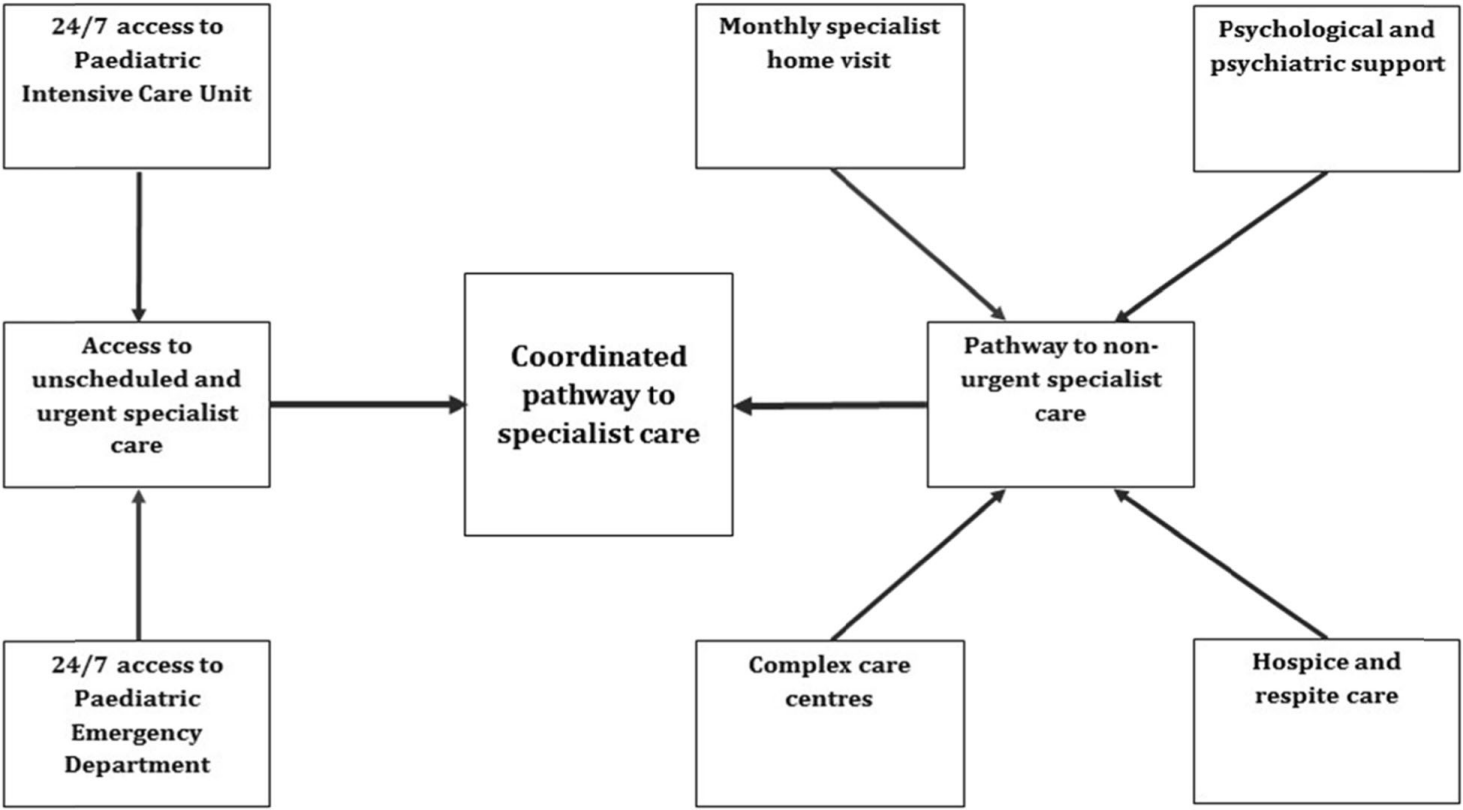
Emergence of global theme coordinated pathway to specialist care

This GT emerged as CAs emphasised the need for access to care for children assisted with LTV, and their families, to specialist services once discharged. Repeatedly there was concern expressed about the inequity in the provision of specialist care, in that pathways to care rarely existed. The GT emerged from two OTs ‘access to specialist unscheduled and urgent care’ and ‘pathway to non-urgent care’ (Fig. 2). Timely access to specialist unscheduled care was identified as critical to the safe and effective care of the child assisted with LTV. In the data returned this was referred to as the ability of families to access a Pediatric Intensive Care Unit (PICU) and a Paediatric Emergency Department (PED) 24/7. The need for a pathway for care, where the parameters for access were clearly understood was identified as critical to support this. In the event of a child having significant respiratory dysfunction immediate access to a PICU was identified as optimum. The OT ‘pathway to non-urgent specialist care’ emerged from four basic themes (BTs): monthly specialist home visit, psychological and psychiatric support, hospice and respite care, and complex care centres. CAs identified a need for a clear pathway for parents to access this support in the community from healthcare, allied healthcare and social care professionals. A number of CAs identified facilitators of optimum non-urgent access as depicted here:

*There is a system called home visitation: medical staff consisting of one intensivist or paediatrician and an enrolled anaesthesiology nurse visits the child on a monthly basis. During this visits not only the tube replacement takes place, but a set of consultations happen. Community caretakers are highly advised to attend, and welcomed, to meet the hospital staff in place.* (Hungary)

There was considerable concern expressed about the absence of respite care available for children on LTV. CAs reported the importance for families to have enhanced access to respite care, either in an unscheduled capacity where there may be a family crisis, or as non-urgent care, to provide respite for the child and family. The establishment of complex care centres was identified as an optimum way forward to enhance holistic care of the child on LTV and their families, to support access and availability to a wide variety of health and social care professionals. An example of this was provided by the CA from Bulgaria who described what was being developed in their country:

*…centres for complex care for children with disabilities and chronic diseases are organisations in which medical and non-medical specialists perform at least one of the following activities: support of the families of children with disabilities and chronic diseases for prescribing and performing early diagnosis, treatment and medical and psychosocial rehabilitation; long term treatment and rehabilitation of children with disabilities and chronic diseases and education of parents for home-care; providing visits of medical specialists for specialised care of children with disabilities and severe chronic diseases, who are looked after at home or at social care residential home; providing specialised palliative care for children.*

### Legal, policy and governance structures

**Fig. 3:**
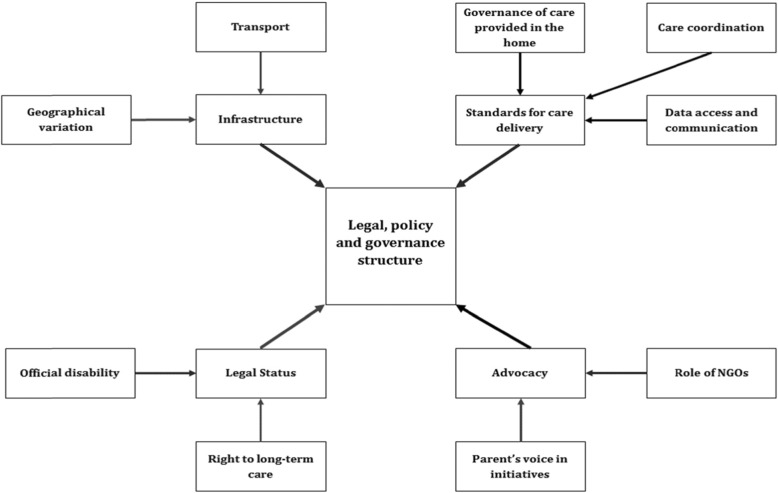
Emergence of global theme legal, policy and governance structures

The GT ‘legal, policy and governance structures’ emerged from four OTs: ‘infrastructure’, ‘standards for care delivery’, ‘legal status’ and ‘advocacy’ (Fig. 3). The OT ‘infrastructure’ emerged from data provided by the CAs on key issues that influence the opportunities for optimum integration of care at the acute community interface. These included geographical variation in access to care, challenges with transport and difficulties with volunteer support. Many countries highlighted concern about inequity in access to care for children on LTV. The CAs reported that resource allocation to support care for children on LTV can vary according to funding in different geographical areas and they also highlighted that access to specialist care, to support the child transitioning to home, varied considerably between urban and rural locations. In some cases this would mean that if a child lived outside an urban centre the family may not be able to access sufficient care for their child to live at home. Challenges were also identified regarding the transport of children assisted with LTV regardless of the child’s geographical location, with ambulances often too small for the ventilation material.

The OT ‘standards of care delivery’ emerged from three BTs: governance of care in the home, national strategy and data access and communication. The issue of governance of care in the home emerged a number of times in comments from the CAs. The majority of CAs reported a number of challenges in governance of care in the home including lack of governance of care agencies and inadequate education of nursing and care staff to care for a child on LTV in the home. This challenge was explained by some CAs by the fact that the number of children on LTV in their country was not very high, and thereby the experience of caring for them was limited. Lack of available clinical expertise in this area was also attributed to an overall shortage of healthcare professionals in many countries.

The majority of CAs suggested that a national strategy on the management of children assisted with LTV was required to address many of the issues raised. However, they highlighted that at present there is a predominant absence of such a strategy or any associated standards of care. The consequences of the absence of such a strategy or standards of care were explained further by a number of CAs. This included no standardised needs-assessment or re-assessment for children and their families. It was also highlighted in a number of CA responses that the child’s legal status was important in facilitating integration of care delivery. This included where the child had official disability status and where there was a legal right established in a country affording right to long-term care for patients with respiratory problems.

The role of non-governmental organisations (NGOs) was identified as a very important facilitator for integration of care at the acute and community care interface, by virtue of the many roles they can play in supporting optimum care delivery. Examples of the supportive role of NGOs included: fundraising to support state funding for care delivery; volunteers and staff from NGOs to organise and deliver care to children assisted with LTV and their families; and acting as advocates for the needs of these families. However, in a number of countries CAs reported no system of volunteers or charity organisations to provide care or support for children on LTV and their families. This was identified as a significant barrier to the provision of care for this population.

## Discussion

The findings show that there is much to be done to enhance the care of children assisted with LTV across the EU/EEA, and indicates that health services are not yet coping with this growing number of children. The data highlights key issues in terms of gaps in care integration. The challenges with integrated care delivery are supported internationally, often due to lack of the fundamental resources that are required to meet the needs of a growing population of children with CCNs and challenges with interagency coordination [17–19]. A further challenge identified is the persistent lack of respite care and the need for an enhanced provision of this service in the home. However, such a service is not a panacea for enhanced care in the community. Specific care risks have been identified for this service, reflecting wider potential problems in the delivery of care at the interface of community and acute care. Communication at the point of the initial referral and ongoing communication pathways, primarily regarding the documentation and handover of care, is a well-recognised challenge in literature on respite care and in wider literature on documentation and safe and effective handover practices [20–23]. Given the variety of statutory agencies, and the reliance on NGOs, in the care of these children, there is the potential of misinterpretation or miscommunication of information.

A further challenge to enhanced integrated care is the current state of advocacy. While the majority of countries reported the involvement of the family in the development of the plan of care prior to discharge, few countries take into account the voice of the families in the development of policies or national frameworks for care delivery to these children. Given the well-documented complexities involved in adapting to the role of the primary care giver for parents, the inclusion of the voices of these families has the opportunity to positively influence care delivery. There is evidence in wider literature on the care of children with CCNs that there are numerous and varied characteristics of a family that need to be understood to support family preparedness for discharge to home that goes well beyond clinical readiness to care. This includes issues relating to language, culture, race and ethnicity [24–26], family structures and support systems [27–29] and capacity for coping [30–32]. The fact that the majority of countries do not currently collect data on the experience of care from the perspective of the parents/guardians or siblings of children assisted with LTV, suggests a persistent paternalistic health service with a limited appetite for identifying and addressing the real needs of the child and family. Understanding these experiences is important to identify specific ways that integration of care can be enhanced in a meaningful way.

There is great complexity in measuring the structures and processes of complex care across 30 countries. We urge caution when interpreting the data as it is always possible that some of the findings might have reflected general issues on LTV as opposed to commentary on the specific scenario presented. Although the use of the scenario afforded the possibility to create a realistic care delivery situation, it is acknowledged that there is concern about the use of hypothetical situations to elicit opinion. Nonetheless, we consider that we received rich contextual data from the CAs, as we relied on a large number of respondents, across a large number of culturally diverse countries. This was supported by the development of a glossary of terms to enhance a consistent understanding of terminology. The perspective of children and families was not included in this part of the MOCHA project and it would be important to include this perspective in further work in this area.

## Conclusions

The growing trajectory of children assisted with LTV in the home places great challenges on healthcare delivery. However, prior to the MOCHA project little was known about the key constituents for effective integration of care for these children and their families. This study has begun to address this knowledge deficit. There are clear challenges for health services to adapt to the changing profile of the needs of the child and the family. While the clinical care in the acute sector has received considerable attention the findings in this study highlight the need for an enhanced focus on the care required following discharge to the community setting and a commitment to supporting care delivery that acknowledges the complexity of contemporary child health issues and the context of the families that become their primary care givers.

## Data Availability

Data sharing is not applicable to this article as no datasets were generated for this part of the MOCHA project.

## Abbreviations

BT: Basic theme
CA: Country agent
CCNs: Complex care needs
GT: Global theme
LTV: Long-term ventilation
MOCHA: Models of Child Health Appraised
NGO: Non-governmental organization
OT: Organizational theme
PED: Pediatric Emergency Department
PICU: Paediatric Intensive Care Unit
US: United States
